# An overview on Sjögren’s syndrome and systemic lupus erythematosus’ genetics

**DOI:** 10.1093/toxres/tfae194

**Published:** 2025-02-23

**Authors:** Ilker Ates, Ulku Terzi, Sinan Suzen, Lalu Muhammad Irham

**Affiliations:** Department of Toxicology, Ankara University, Faculty of Pharmacy, Emniyet Distr, Degol Str, No. 4, 06560 Yenimahalle, Ankara, Turkey; Department of Toxicology, Ankara University, Faculty of Pharmacy, Emniyet Distr, Degol Str, No. 4, 06560 Yenimahalle, Ankara, Turkey; Department of Toxicology, Ankara University, Faculty of Pharmacy, Emniyet Distr, Degol Str, No. 4, 06560 Yenimahalle, Ankara, Turkey; Department of Toxicology, Ahmad Dahlan University, Faculty of Pharmacy, Prof. Dr. Soepomo, S.H., Street, Warungboto, 55164, Yogyakarta, Indonesia

**Keywords:** Systemic lupus erythematosus, Sjogren’s syndrome, genetics, autoimmune diseases

## Abstract

Major autoimmune rheumatic disorders, such as systemic lupus erythematosus and Sjögren’s syndrome, are defined by the presence of autoantibodies. These diseases are brought on by immune system dysregulation, which can present clinically in a wide range of ways. The etiologies of these illnesses are complex and heavily impacted by a variety of genetic and environmental variables. The most powerful susceptibility element for each of these disorders is still the human leukocyte antigen (HLA) area, that was the initial locus found to be associated. This region is primarily responsible for the HLA class II genes, such as DQA1, DQB1, and DRB1, however class I genes have also been linked. Numerous genetic variants that do not pose a risk to HLA have been found as a result of intensive research into the genetic component of these diseases conducted over the last 20 years. Furthermore, it is generally acknowledged that autoimmune rheumatic illnesses have similar genetic backgrounds and share molecular pathways of disease, including the interferon (IFN) type I routes. Pleiotropic sites for autoimmune rheumatic illnesses comprise TNIP1, DNASEL13, IRF5, the HLA region, and others. It remains a challenge to determine the causative biological mechanisms beneath the genetic connections. Nonetheless, functional analyses of the loci and mouse models have produced recent advancements. With an emphasis on the HLA region, we present an updated summary of the structure of genes underpinning both of these autoimmune rheumatic illnesses here.

## Introduction

**Fig. 1:**
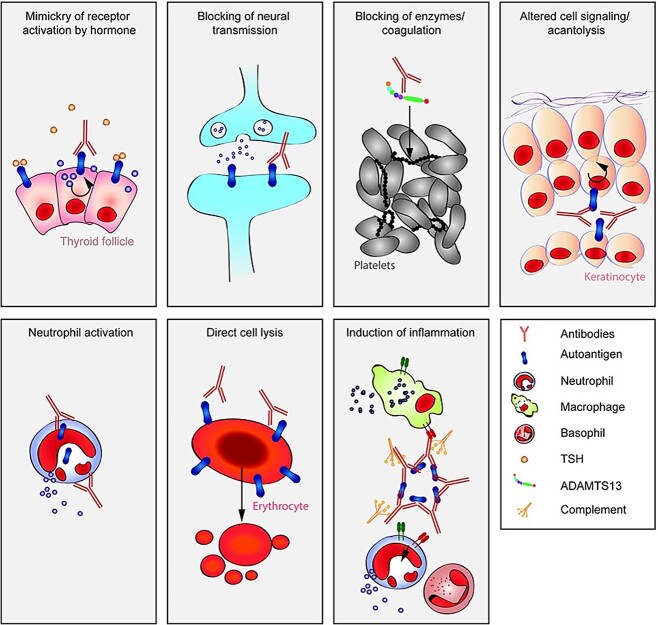
Pathology generated by autoantibodies is caused by many routes.^4^

Once the immune system’s capacity to tolerate self-antigens from its own organism is diminished, it can cause autoimmunity, which progressively damages one or more tissues. A complex set of disorders with over 80 immune-induced inflammatory syndromes and a wide variety of overlapping signs and symptoms and presentations are the outcome of this improper immune response.^1^ Despite the modest frequency of each condition individually, together they impact around 5%–9.4% of the overall population, which suggests a significant health and socioeconomic impact given their chronic and debilitating character.^2^^,^^3^ In this analysis, we concentrate on two primary conditions that serve as emblematic instances of autoimmune rheumatic diseases: Sjögren’s syndrome (SS) and systemic lupus erythematosus (SLE). One characteristic of these illnesses is the existence of autoantibodies (Fig. 1).

Numerous different clinical symptoms, from mild skin erythema to serious harm to neurological system, renal inflammation, and kidney failure, are linked to SLE, the model for systemic autoimmune diorders. SLE is defined by any signs of autoantibodies contrary to nuclear antigens. SS is characterized by tissue damage to exocrine glands (mostly the lacrimal and salivary glands) and lymphocytic infiltration, which causes mucosal membranes to become dry. Nonetheless, the disease’s range is broad; it can affect the gastrointestinal, vascular, musculoskeletal, hematologic, pulmonary, renal, dermatological and neurological systems as well as be an organ-specific condition.^5^ SS can manifest in two ways: as primary SS (pSS) or secondary SS (sSS), when it coexists with other autoimmune illnesses as rheumatoid arthritis (RA), SLE, systemic sclerosis (SSc) or Hashimoto’s thyroiditis, along with others.^5^

Our understanding of the pathophysiology of these disorders is limited due to their varied nature. The etiology of autoimmune rheumatic illnesses is complex, involving both hereditary and environmental variables that impact the disease’s development and course. In genetically sensitive individuals, environmental factors like smoking, UV radiation exposure, medicine, hormone fluctuations, and recurring infections have been firmly linked to these diseases.^6^ For example, Epstein–Barr virus infection is believed to be linked to a number of autoimmune rheumatic disorders. The SLE patients with antibodies against the Smith (Sm) protein provide the strongest evidence for this link,^7^ which supports the molecular mimicry theory.

From a genetics perspective, twin and family studies provided us with an early grasp of the genetic background. For instance, it has been noted that monozygotic twins have greater SLE concordance rates than dizygotic twins.^5^ Additionally, twin studies have shown that autoantibody production is highly concordant in SSc patients,^8^ and they have calculated that the variation in phenotype described by the a genetic component or RA heritability, is roughly 60%.^9^ Furthermore, there has been evidence of high family aggregation associated with these ailments, with it being rather frequent for individuals from the same family to suffer from multiple autoimmune rheumatic disorders.^10^ Although early researches indicated a significant role for the hereditary component in the onset of many disorders, heredity is a complicated phenomenon that is challenging to understand.

The scientific world has given the study of genetic background significant attention in recent decades, and the amount of large-scale studies on genetic associations being published is growing exponentially. Hundreds of loci that are strongly linked to autoimmune rheumatic disorders have been uncovered using these methods with remarkable effectiveness.^11^ Many of these loci are found to be shared among various diseases. We will update our current understanding of the genetic terrain of the two main autoimmune rheumatic diseases—SLE and SS—in this review. Given that it carries the most powerful susceptibility factor associated with autoimmune rheumatic illnesses, particular emphasis will be paid to the human leukocyte antigen (HLA) area.

## Associations of human leukocyte antigens

The majority of autoimmune rheumatic illnesses, such as SLE, SSc, RA, and SS, were found to be susceptible to the HLA region initially.^12–14^ This remains the most prevalent genetic susceptibility for these illnesses and has been repeatedly validated by numerous independent investigations. The HLA region’s complexity is being untangled by the advent of novel innovations in the era of genomics, including fine-mapping and particular imputation algorithms. These technologies are revealing associations between novel alleles and their relationships to individual amino acids that are crucial for understanding the composition of the HLA’s antigen-presenting groove. Proteins produced by the HLA class II gene are necessary for the presentation of antigen to CD4 + T helper cells, that starts the immune response that leads to the production of specific antibodies. These days, it is commonly acknowledged that distinct alleles of the HLA class II genes, namely DQA1, DQB1, and DRB1, are linked with SLE and SS. Furthermore, a link with HLA class I has been discovered, recommending a function for CD8 + T cell biology in the pathophysiology of autoimmune rheumatic diseases.

The haplotype that includes the HLA alleles of B8*0801, A2*0101 and DRB1*03:01 as well as the DQB*0201 and DQA*0501 was the main genetic connection in SLE.^15^ The class III HLA genes, which are also linked to the complement genes C2 and C4, TNFa (TNF) and lymphotoxin (LTA), besides other genes, are contained inside these class I and class II HLA genes. Several other haplotypes have also been linked to their DRB1 alleles, such as the European (EUR) DRB1*15:01 (DR15 haplotype),^16^ which is the most common, followed by DRB1*08:01 (DR8) and. DRB1*03:01 (DR3). Replication has been specifically reliable for the DR2 haplotype, which contains the traditional alleles DQB1*06:02, DRB1*15:01, DQA1*01:02,^15^ and DRB5*01:01.^17^ It’s interesting to note that protecting alleles or haplotypes were Native American, while HLA risk alleles had a European origin in a sample of people with mixed Amerindian and European ancestry, according to local ancestry analysis.^18^ African Americans (AA) and European Researchers conducted a joint analysis of HLA risk alleles, and the results indicated that the narrow, separate peaks in the AA data correlated with the far-reaching signals related to SLE in EUR. Independent relationships peculiar to a population were found. Even yet, the vast majority of susceptible alleles showed substantial consistency in the pattern of the allele impacts and similarity to the European HLA SLE connection, despite the population’s high degree of diversity.^19^ Likewise, in Korean and various East Asian cohorts, DQB1*06:02 and DRB1*15:01 were linked to SLE risk.^16^^,^^20^ Certain The HLA alleles have been connected to the different autoantibody specificities seen in SLE, especially in the case of anti-SSA/Ro antibodies with the DR3 (as represented by DRB1*0301) or DR2 (as represented by DRB1*1501) haplotypes.^21^ In an extensive East Asian sample, the role of particular autoantibodies and certain amino acid remaining materials in relation to the likelihood of forming SLE was examined more recently.^22^

In recent times, there has been a slight alteration in the HLA genetic association. Upon analysing the heterogeneity of SLE, it was observed that the group of SLE cases with the interferon (IFN) signature—that is, gene expressions induced by type I IFN—in their blood cells exhibited the strongest HLA class II genetic association. In contrast, the IFN signature was almost absent in the other groups.^23^ Patients with autoantibodies that resembled SS, such as SSA and/or SSB, were also included in this group. Focused next-generation genome sequencing of the HLA area revealed an interesting haplotype containing regulatory polymorphisms linked to variations in the HLA class II molecules’ gene expression.^24^ Moreover, a recent study shown that DRB1 interferon-dependent gene expression was linked to hypomethylation of STAT1 and DRB1.^25^

Last but not least, early research on Sjögren’s syndrome also found significant HLA associations.^26^ These correlations were observed in individuals with anti-SSA or anti-SSB antibodies linked with the DRB1*0301 haplotype (DR3), which is strikingly comparable to what we previously addressed in SLE. These connections were also observed in patients with other conditions. Since the existence of anti-SSA and SSB antibodies is a key component of Sjögren’s syndrome, these correlations are actually noticeably higher in this condition.

### Identification of non-HLA associations by extensive genetic research

Over the past ten years, the introduction of new genotyping techniques and statistical tools has significantly increased our understanding of the genetic composition of autoimmune rheumatic illnesses. Strong large-scale genetic analysis, like genome-wide association studies (GWAS) and Immunochip research, are now feasible thanks to these developments. Both methods concentrated on examining typical genetic variations in sizable cohorts of cases and controls, mainly single nucleotide polymorphisms (SNPs). GWAS employ a hypothesis-free approach to assess genetic associations across the entire genome, whereas the Immunochip is a specialized high-density array specifically intended for fine-tuning regions associated with various immune-mediated disorders.^27^ Recently, hundreds of loci linked to an increased risk of SLE and SS have been found by numerous large-scale genetic investigations.^28^ These research have documented genetic predispositions to these diseases.

### Systemic lupus erythematosus

**Fig. 2:**
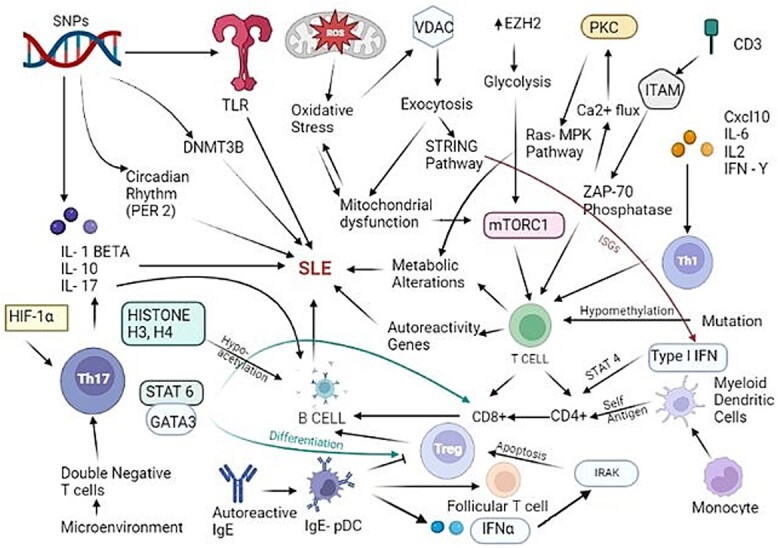
Established processes and their interplay in the modulation of SLE.^32^

Adult cases of sporadic SLE were long believed to be the only cases that qualified for this diagnosis, despite the fact that SLE can also strike very young children. It is now acknowledged that systemic lupus erythematosus is the cause of cases of complement C1s and C1r deficits in young people.^29^ For pediatric SLE, these complement deficits have been firmly established as a hereditary etiology; however, adult occurrences of the disease do not follow this pattern. It was not up to recently that it became apparent that a deletion in C4 could actually be involved, along with the presence of copy number variants in some cases,^30^^,^^31^ not as a major deficiency but instead as another locus that increases the risk of developing the disease. Numerous studies^30^^,^^31^ searched for C4 deficiencies. Low levels of C4 are sometimes seen in SLE patients, and they are typically linked to increasing inflammatory processes as the disease progresses. However, disease activity rather than a hereditary deficit may be the reason of this decline. Fig. 2 illustrates the established processes and their interplay in the modulation of SLE.

A traditional analysis conducted on a spesific European group, the Finns, did estimate a heritage of 66% ± 11% for SLE,^33^ showing that more than half of the heritability for SLE from GWAS reported at the time was still missing. In spite of this, it is challenging to determine the total heredity of complex illnesses. These heritability estimates often only take into account common genetic variations from the GWAS and ignore uncommon variants. Furthermore, the variability of the illness could make it difficult to determine the true genetic makeup of some people.

In the end, the recent GWAS found many entirely new associations in addition to known ones, like the FcGR family of genes.^34^^,^^35^ Aside from HLA, the following genes showed the most noteworthy and frequently replicated associations: interferon regulatory factor 5 (IRF5), BLK, ITGAM, BANK1, IRF7 and IRAK1/MECP2. Among them, IRF5 has been the gene most frequently replicated. Studies tried to determine the functional processes underlying the genetic association and the location of the association within the gene or gene surrounds after the identification of TYK2^36^ and this one.^37–40^ First noted in rheumatoid arthritis,^41^ the genetic correlation with PTPN22 was later investigated in SLE.^42^ The functional studies came next. Another candidate gene was STAT4,^43^ whose genetic relationship was clearly verified across several populations and research. Nevertheless, the genetic association consistently suggested alterations in STAT1 gene expression, as STAT1 and STAT4 appeared to be intergenic. An examination based on fine-mapping and expression quantitative trait locus (eQTL) analysis supported the impact of the STAT4-located SLE risk rs11889341 mutation on the expression of neighboring STAT1, but not on the increase in STAT4 expression in B cells.^44^ Recent studies have shown that T cells that produce IFN-g in response to interleukin (IL)-12 have changes in STAT4 expression.^45^ Given STAT4’s known role in the Th1–Th2 differentiation induced by IL-12, this result was expected.

The initial GWAS published in SLE included a single lupus patient from each of six families with several SLE cases, all of which were female. The genes PXK, ITGAM and KIAA1542, which relate to IRF7, were the main ones identified by this GWAS, and it also confirmed a few more loci.^46^ Nearly concurrently, a second GWAS revealed the area including C8orf13-BLK and ITGAX (near ITGAM) as genes of susceptibility for SLE.^47^ Lastly, it was revealed concurrently, but independently, of both the large GWAS^48^ and ITGAM,^49^ that BANK1 was associated with SLE. The gene BANK1, which is mostly expressed in B cells, has been linked to at least two separate populations.^48^^,^^50^^,^^51^ Researches conducted in vivo and in vitro have shown BANK1’s involvement in the TLR7 signaling cascade.^52^^,^^53^ Recently, a thorough analysis of BANK1 was released.^54^

The only significant genetic relationship linked to a clinical measure of lupus nephritis is that of ITGAM, TNIP1, and STAT4.^55–57^

Following these initial research studies on candidate gene (e.g. IRF5, PTPN22, BANK1, STAT4, ITGAM) and the primary GWAS, numerous additional studies have been conducted to confirm all genetic associations, fine-map these, and discover a plethora of other genes, many of which are connected to other autoimmune rheumatic illnesses.

As of right now, there are a lot of risk loci, many of which are found in genes that impact the functioning of innate immunity or B cell function. Nevertheless, a large number of loci are found in intergenic regions where no obvious association exists with any gene; as a result, it is unknown how precisely gene expression or activity is regulated. Remarkably, a recent study discovered areas near SLE susceptibility genes that may be managed by the Epstein–Barr virus’s transcription factor EBNA2, indicating that this widespread virus directly controls the genes for SLE as well as other autoimmune diseases.^58^

Numerous investigations, such as those examining miR146a, have improved our understanding of the functioning of putative genetic risk variations and fine-mapped many of those found in SLE. A first investigation found variations in the microRNA linked to the risk allele’s gene expression.^59^

Later, Tang and coworkers explained how MiR146a targeted significant signaling molecules to increase the expression of the type I IFN route.^60^ More recently, it was discovered that the associated SNP of MiR146a tags a the distal booster that communicates with the Mir146a promoter,^61^ which helps to unravel the regulatory mechanisms underlying the SLE risk alleles. This is but one illustration of how further information could be obtained from the work utilizing cutting-edge methods like genome editing or 3D chromatin structure research.

Studies have also been done on the female sex bias in SLE. The Cxorf21 locus is one such instance. This locus has been found to evade X-inactivation, which raises the chance of lupus in women.^62^ TLR7, a significant lupus gene that has previously been shown to evade X-inactivation, exhibits a similar behavior.^63^ Additional research on Cxorf21 has revealed that female monocytes express this open reading frame more than male monocytes do, and that there is a strong correlation between it and TLR7.^62^ More recently, Brown et al. found a gain-of-function mutation in a girl with lupus, as part of an effort to find unusual mutations causing lupus in pediatric instances. This discovery defined many irregularities this de novo mutation had on the illness etiology.^64^ Crucially, the mutation in animal models caused the sickness due to the amino acid sequence harboring the mutation is mostly conserved across species. It was demonstrated, concentrating on the function of B cells and the impact of TLR7, the most significant B cell population implicated in the disease advancement was age-associated B cells.

There are noteworthy findings from the investigation of genetic risk among ancestries. Three ethnic groups— Hispanic, African-American and European—Had their primary risk alleles for SLE more precisely established by a significant trans-ancestral mapping study. The probability of developing lupus is increased by the accumulated existence of risk alleles.^65^

### Sjögren’s syndrome

**Fig. 3:**
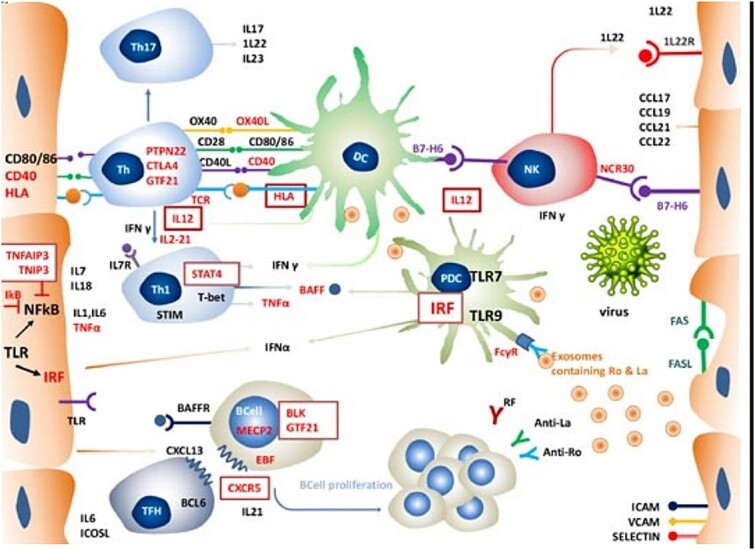
SGEC and immune cells’ roles in the pathophysiology of Sjogren’s syndrome.^66^

Previously thought to be passive observers, salivary gland epithelial cells (SGEC) appear to be the center of pathogenetic events in SS. Many inflammatory cytokines, including TNFα, IL-1, and IL-6, are produced by SGEC and are essential for immune responses that are innate as well as adaptive. This includes cytokines such as interferons that are involved in B cell activation, Th1, Th17, and T follicular helper cell response. Immune cell interactions resulting in salivary gland inflammation, lymphocyte proliferation, and occasionally ectopic germinal center formation cause the development of SS, as seen in Fig. 3. These proinflammatory chemicals are the end consequence of their creation.^66^

Besides the HLA class II region’s highest connections, studies on candidate genes have shown a number of additional loci for SS, including IKZF1, MTHFR and TNFAIP3. The HLA class II area has been found to have the strongest connection in studies employing GWAS in cases that are both Asian and European-derived.^67–69^ Other genes that are already linked to SLE, like IRF5, STAT4 and BLK-FAM167A, have also been linked, while other genes for SS, IL12A, and OAS1 have been linked.^70^ OAS1’s isoforms were examined in an intriguing study, and the results revealed altered gene expression in cases compared to controls. The X-chromosome gene CXorf21 is another gene that is shared by SLE and SS. It may act as a mediator between the female bias and the X-chromosome dosage effect. Anti-SSA and/or anti-SSB antibody-positive patients differ genetically from individuals who test negative against these antibodies; the former have genetic links not only to the HLA but also to IRF5 and a newly identified gene termed GOT1.^71^

### Genetically related risk factors

As was previously indicated, autoimmune rheumatic disorders have been shown to share molecular underpinnings of disease pathogenesis, as evidenced by the elevated levels of comorbidities and familial clustering among them.^1^^,^^72^ The observation of pleiotropic effects in a significant number of loci and genes linked to these disorders provides more evidence for a shared genetic basis. They share a sizable portion of the loci and genes connected with every particular disease in this review.

Through straightforward study comparison, the initial pleiotropic loci for immune-mediated disorders, like the HLA area, were discovered.^73^ TNIP1, IRF5, STAT4, and other renowned shared risk loci are examples of autoimmune rheumatic disease loci. Cross-phenotype studies, a method that combines genetic data from many diseases, have proven to be an effective technique to uncover similar pathways in the pathophysiology of different conditions and find new pleiotropic loci. Furthermore, by using this approach, the statistical ability to identify connections with low-effect genetic variations is increased. Recent publications of multiple cross-phenotype investigations have contributed to our comprehension of their shared genetic basis. For instance, it is important to highlight two comprehensive cross-phenotypes using GWAS and Immunochip data, respectively, from sizable cohorts of people with RA, SSc, and SLE, among other autoimmune rheumatic diseases, as a result of this technique.^74^^,^^75^ New susceptibility loci were found, including NAB1, that has been connected to the type I IFN signaling pathway. In a recent study, rare and harmful germline variants predisposing to SLE, RA, and SS were found utilizing whole-exome sequencing of 31 families, highlighting similar T cell–activating genes and supporting a shared etiology in certain autoimmune rheumatic disorders.^76^ Taking into account everything said above, reclassifying these illnesses according to molecular patterns may aid in novel biomarker discoveries for improved diagnosis and prognosis.^77^ Accordingly, three distinct groups with particular molecular patterns corresponding to lymphoid, inflammatory and IFN clusters were found in an unsupervised clustering study of the total blood methylome and transcriptome information from seven autoimmune rheumatic disorders.^23^

Furthermore, enormous amounts of genome-wide genomic data give useful details to analyze differences as well as similarities among these disorders through the use of novel statistical techniques such genetic risk scores and linkage disequilibrium score regression. Using these methods, for example, has revealed that RA has a positive genetic connection with SLE and a detrimental genetic correlation with schizophrenia, which is congruent with epidemiological results.^78–80^ Surprisingly, a recent investigation proposed that the application of genome-wide genomic data could aid in the differentiation of inflammatory arthritis-associated disorders such gout, RA, SLE, spondyloarthropathy, and psoriatic arthritis.^81^

### The post-GWAS investigations to ascertain the objectives of the genetic associations

Even while our knowledge of the genetic makeup of autoimmune rheumatic diseases has greatly improved recently, it is still challenging to convert the findings of extensive genetic research into biological understandings. In certain instances, a putative causal variant is discovered and the discovered polymorphisms describe the genetic connection. This is primarily the case when there are non-synonymous changes in coding exons that alter the expression or function of the protein and there aren’t any other possible variations that might be in linkage disequilibrium and could also account for several of the functional abilities. The most typical scenario, however, is that GWAS/Immunochip results reliably identify genomic areas linked to disease, typically with tens to hundreds of variations that are statistically identical because of the linkage disequilibrium pattern. Determining the causal relationship between these variations presents another difficulty. Ninety percent of the genetic variants linked to complicated disorders, including autoimmune rheumatic diseases, have been found to map to non-coding regions. As a result, the effects of these variants cannot be directly connected to the function of the target proteins or the molecular mechanisms of the cell types they affect.^82^ This is because it has been shown that the majority of variants associated with disease are located in genomic regulatory regions. New methods are therefore quickly developing in the arena to address these constraints. One effective tactic, for instance, has been shown to be the combination of data from genomics with other omic details, such as transcriptomic and epigenomic data. As this review’s earlier sections have explained, recent research has carried out functional fine-mapping in cell types and tissues by integrating with chromatin interaction maps or epigenetic features. The results have indicated the best nominee gene and putative molecular pathways that may be important for the illness.

Always be open to other options in any case, since newly discovered variants that haven’t been genotyped or sequenced might also exhibit functional changes that go well with other changes or are present in populations of patients. Moreover, mouse models are offering significant new information about the medicinal and clinical applications of genetic knowledge. Here, we will provide an in-depth functional analysis of a few pleiotropic gene examples for the illnesses included in this review.

#### TNIP1

Numerous autoimmune illnesses, including systemic sclerosis, rheumatoid arthritis (RA), psoriasis, and SLE have been linked to TNIP1 gene polymorphism.^83–89^ Furthermore, a correlation was observed between it and seropositivity for anti-Ro/SSA and anti-La/SSB autoantibodies in SS.^67^ Conversely, the TNFAIP3 gene has been linked in GWAS studies to pSS and has also been linked in reports to disorders like SLE and RA.^90–93^ There have been reports linking pSS to allele variants of the TNFAIP3 gene.^94^^,^^95^ Scientific investigations have identified a relationship between the TNIP1 rs17728338 polymorphism and a number of inflammatory and autoimmune illnesses, including psoriatic arthritis and psoriasis. The TNIP1 rs17728338 polymorphism and SS have been the subject of few investigations. Nordmark et al.^88^ discovered that TNIP1 polymorphisms are associated with primary SS that is antibody-positive. Furthermore, we discovered that minor variant in the TNIP1 polymorphism increased the risk of SS patients by approximately five times when compared to the control group.

ABIN1, which is involved in the regulation of NF-kB, an essential inflammatory regulator, is encoded by the tumor necrosis factor-a (TNFa)-induced protein 3-interacting protein 1 (TNIP1) gene. Prior to the discovery of two unique haplotype effects close to the gene’s promoter, which were associated with lower levels of gene expression in EBV-transformed cell lines,^96^ a TNIP1 variant has been persistently linked to lupus nephritis, as well as SSc, RA, and SS. A coding missense variant (rs2233290) in one of the haplotypes results in the ABIN1 mutation P151A. ABINN1 decline in functionality has significant effects on the course of SLE development. By attaching to NEMO, a crucial part of the IKK complex, ABIN1 blocks NF-kB activation of transcription by stopping IkB-a from being phosphorylated and degraded. Mutant D485N, a transgenic mouse devoid of ABIN1’s inhibitory and ubiquitin binding activities, forms spontaneous lupus-like symptoms and nephritis.^97^

#### DNASE1L3

Highly expressed in DCs, DNASE1L3 is a Ca2+/Mg2 + −dependent endonuclease that has a crucial functional role in controlling autoimmune reactions to chromatin and self-DNA. In mice as well as humans, autoimmune disorders arise from a deficiency of DNASE1L3.^98^

SLE was initially identified in members of a Saudi Arabian population with widespread endogamy who carried a loss-of-function mutation.^99^ Subsequent research has confirmed its involvement in other illnesses, such as RA and SSc. Similar to DNASE-1, DNASE1L3 is a secreted nuclease that breaks down chromatin DNA. When Dnase1L3 is deleted in mice, the plasma DNA fragments abnormally, leading to an enhancement in small DNA molecules (less than 120 bp). This demonstrated a function for this molecule in maintaining plasma DNA homeostasis and was linked with anti-DNA antibody levels.^100^ More recently, it was demonstrated that a missense mutation (R206C) results in a deficiency in DNASE1L3’s protein production, increasing the risk of SLE and maybe other disorders linked to it.^101^ Paç Kisaarslan and her colleagues^102^ reported a case of monogenic lupus involving a homozygous mutation of c.537G > A (p. Trp179Ter) in DNASE1L3 in a recent investigation. Five cases of hypocomplementemic urticarial vasculitis with the DNASE1L3 mutation within two families were reported by Ozcakar et al.^103^

#### IRF5

IRF5 is a multi-domain 60–63 kDa protein comprising an amino-terminal DNA-binding domain, an association domain, and a transcriptional co-activator binding domain at the C-terminus.^104^ All of these domains are essential for this gene’s transcriptional function, which happens after initial activation by serine phosphorylation, dimerization, nuclear translocation, and binding to gene promoters, among other transcription factors. Being a gene associated with the interferon type I pathway, the IRF5 has been considered a great contender. Thus, genetic differences in type I interferon-related genes confer risk for several autoimmune rheumatic illnesses; SLE and SS have been linked to IRF5, in particular. Overactivity of this important route has been consistently noted in these disorders, particularly in SLE and SS.^105^^,^^106^ Because of their possible involvement in the TLR7 pathway, IRF5 and many of its related genes are gaining more and more attention. Genes like IRF5, DNASE1L3, and TNIP1 appear to be essential components of the type I IFN pathway in SLE (and potentially SS).^107^

IRF5’s primary function in SS is to produce Type-I IFNs and proinflammatory cytokines such IL-12, IL-17, IL-23, and TNFα.^108^ IRF5 is linked to pSS in patients of all examined ancestries, with the most significant association being a CGGGG insertion/deletion (indel) polymorphism in the promoter region of the gene.^69^ A further risk haplotype resulting from mutations spanning both IRF5 and TNPO3 is exclusive to European ancestry.^109^ In fact, a study assessing the function of IRF5 in SLE discovered that blocking IRF5 hyperactivation lessens the severity of the illness and postpones its beginning.^110^ Peptide mimetics that could attach to IRF5, of which N5–1 in particular exhibited a considerable affinity, were produced in this study. It’s interesting to note that when TLR agonist stimulation occurs, the peptide may penetrate the cells and prevent IRF5 from moving to the nucleus. This peptide provided protective effects when used in vivo in a lupus-prone animal model. Human SLE patients have circulating levels of IRF5, and more significantly, IRF5 levels are measurable in plasma and may be used as an indicator for disease.^111^

## Conclusion

This review outlines how, over the past few decades, the development of new techniques and global cooperative efforts have made it possible to conduct extensive genetic studies that have analyzed billions of SNPs in many thousands of individuals, greatly expanding our understanding of the genetic makeup of complex autoimmune rheumatic diseases. Numerous disease-associated loci have been found by these investigations, which has advanced our knowledge of the genetic pathways underlying autoimmune rheumatic disorders. The scientific community is still dealing with issues, though, and more work needs to be done. To begin with, bigger sample numbers are still required in order to achieve the statistical power required to identify variations with mild and minimal impact, particularly for illnesses like SS for which few loci have been found. International cooperation is thus essential to the ongoing discovery of new information. The primary obstacle to be addressed is how to use these findings in clinical settings, as we have already discussed. In the future, the identification of biomarkers that enable advancements in early diagnosis, categorization, and the continued efforts to identify the causative mutations will, therefore, enable therapy, the genes and molecular mechanisms driving the pathophysiology associated with these disorders.
